# Hypereosinophilic Syndrome: A Case of Fatal Löffler Endocarditis

**DOI:** 10.1155/2016/2359532

**Published:** 2016-01-21

**Authors:** Mario Enrique Baltazares-Lipp, Juan Ignacio Soto-González, Carlos Manuel Aboitiz-Rivera, Héctor A. Carmona-Ruíz, Benito Sarabia Ortega, Ruben Blachman-Braun

**Affiliations:** ^1^Departamento de Hemodinamia y Ecocardiografía, Instituto Nacional de Enfermedades Respiratorias “Ismael Cosío Villegas”, 14080 Mexico City, Mexico; ^2^Facultad de Ciencias de la Salud, Universidad Anáhuac México Norte, 52786 Estado de México, Mexico

## Abstract

Hypereosinophilic syndrome (HES) is a rare disorder with unknown global prevalence, barely reported in Hispanic population, and characterized by persistent eosinophilia in association with organ dysfunctions directly attributable to eosinophilic infiltration. Cardiac involvement may be present in 50 to 60% of the patients. This is known as Löffler endocarditis. We present a case of a 36-year-old Hispanic man with signs of heart failure. Laboratory studies showed eosinophilia (23,100/*μ*L). Thoracic computer tomography showed bilateral pleural effusion and a large left ventricular mass. Transthoracic echocardiography showed left ventricle apical obliteration and a restrictive pattern. Pulmonary angiography demonstrated a thrombus in the lingular and middle lobe. Despite treatment, the patient deceased seven days after admission. Autopsy confirmed the diagnosis of Löffler endocarditis.

## 1. Introduction

Hypereosinophilic syndrome (HES) is a rare disorder with unknown global prevalence, barely reported in Hispanic population [1]. HES is traditionally defined as persistent eosinophilia with more than 1500 cells per microliter for at least six months, which remains unexplained despite a comprehensive evaluation, in association with organ dysfunctions directly attributable to eosinophilic infiltration [2, 3]. In 1936, Löffler described the first cases of HES with cardiac involvement (Löffler endocarditis) [4].

Cardiac involvement is secondary to the myocardium and endocardium damage due to the eosinophils infiltration and degranulation, which release toxic proteins, thus creating tissue inflammation and later fibrosis. Löffler endocarditis is present in 50 to 60% of HES cases; this is usually characterized by endocardial thickening, atrial dilatation, a restrictive pattern in Doppler echocardiography, and ventricular obliteration by an echogenic material, suggestive of fibrosis or thrombosis frequently located in the apical region of the left and right ventricles. HES can present a slow or a rapid (acute) progression, this last one especially when the heart or central nervous system is involved. The prognosis is poor, and death is usually due to congestive heart failure, often with associated renal, hepatic, or respiratory dysfunction [5–7].

In this paper, we present one of the few reported cases of Löffler endocarditis in Hispanic population in addition to a clinical, radiological, tomographic, echocardiographic, and pathological correlation with literature review of this rare entity.

## 2. Case Presentation

A 36-year-old Hispanic male admitted with persistent symptoms of congestive heart failure that began 12 days before admission and persists despite standard medical treatment. During physical examination, he presents atypical chest pain, progressive dyspnea, orthopnea, palpitation, productive cough, and fever. Physical examination revealed normal blood pressure (110/70 mmHg), tachycardia, tachypnea, elevated jugular vein pressure, and congestive hepatomegaly, in functional class III according to the New York Heart Association (NYHA). Cardiac auscultation revealed a third heart sound as well as mitral and tricuspid holosystolic murmurs; crackles were heard in both lungs and edema was observed in both legs.

**Figure 1:**
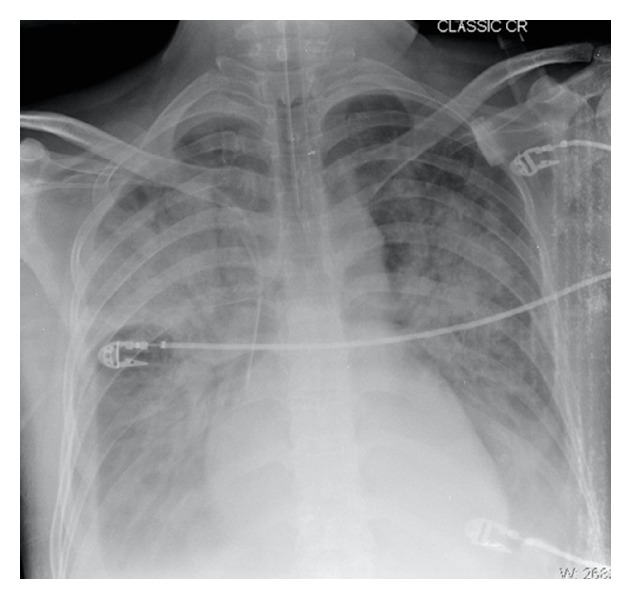
Anteroposterior chest radiography, which shows diffuse pulmonary congestion with bilateral pleural effusion.

**Figure 2:**
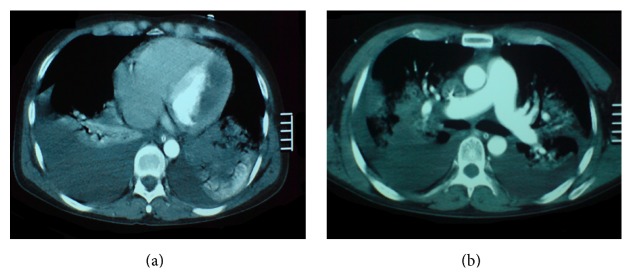
Chest computer tomography with contrast showing (a) a thrombus in the left ventricle and (b) bilateral pleural effusion and dilated main pulmonary artery and its branches.

**Figure 3:**
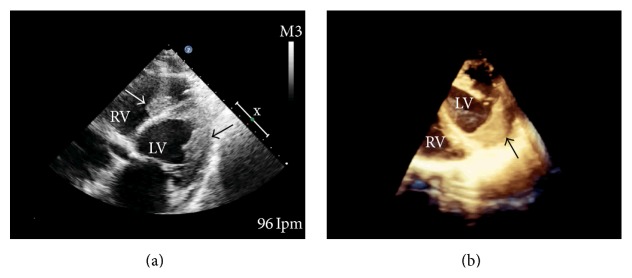
(a) Transthoracic echocardiogram in a modified apical four-chamber view, showing the left ventricular apex with obliteration and the lateral wall thickened by an image suggestive of a thrombus (black arrow) in the right ventricle and image suggestive of a smaller thrombus attached to the septum (white arrow). (b) Transesophageal echocardiogram with a 3D reconstruction, showing a thrombus in the left ventricle (black arrow). LV = left ventricle; RV = right ventricle.

Chest radiography demonstrated pulmonary congestion with bilateral pleural effusion and cardiomegaly (Figure 1). Laboratory test revealed a marked leukocytosis (23,100/*μ*L) with hypereosinophilia (59%, 13,360/*μ*L). Computed tomography of the chest showed bilateral pleural effusion and a large left ventricular mass (Figure 2). The transthoracic echocardiogram showed moderate tricuspid and mild mitral regurgitation with normal left ventricular dimensions and systolic function; left ventricular filling was reduced because of endocardial thickening together with a large homogeneous mass at the apex that occupied 50 to 65% of the left ventricular cavity (Figure 3). Echocardiographic Doppler detected restrictive-type diastolic filling an *E*/*A* ratio greater than 2. The echocardiography also revealed another mass in the right ventricle. A coronary angiography was performed and found no significant coronary artery disease; pulmonary angiography demonstrated a thrombus in the lingular and middle lobe.

**Figure 4:**
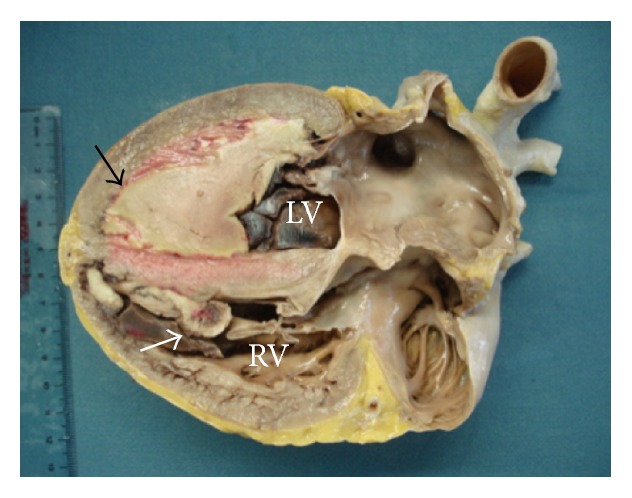
Patients heart, showing a thrombus located in the left ventricle (black arrow), with an endothelium cover, and myocardial infiltration. Additionally, a right ventricular thrombus (white arrow) attached to the septum and covered by endothelium. In addition, there is right ventricular thickness of the free wall. LV = left ventricle; RV = right ventricle.

An endomyocardial biopsy was performed; however, pathologic examination of the obtained specimens revealed mainly thrombus with some necrotic tissue. Despite the biopsy results, a diagnosis of endomyocardial fibrosis secondary to HES was made, on the basis of the imaging, clinical, and laboratory findings, and other secondary causes of hypereosinophilia were ruled out. Despite the team effort and adequate treatment, patient deteriorates to NYHA class IV and died seven days after admission. Then, autopsy was done which confirms the diagnosis of Löffler endocarditis (Figure 4).

## 3. Discussion

Although the real epidemiology of HES is unknown, it is estimated that 90% of patients are men; the majority of the cases occur between 20 and 50 years of age, with a peak in the fourth decade of life [3]. The clinical manifestations of HES are markedly heterogeneous with a wild clinical spectrum from a completely asymptomatic to a life-threatening condition; this pathology can involve many organs and systems such as skin, lungs, nervous system, gastrointestinal tract, kidneys, and heart; therefore the diagnosis could be a challenge [3, 4]. The major morbidity and mortality in HES patients are cardiovascular complication, which is found in 40 to 50% of the cases [3].

Löffler endocarditis presents with extensive infiltration of the ventricular endocardium by eosinophils, with degranulation and arteriolar necrosis with subsequent endomyocardial fibrosis. The inflammatory changes result in thrombus formation, in this case occupying both ventricular cavities, with impairment of diastolic filling and a resultant restrictive cardiomyopathy [8, 9]. The clinical presentation was consistent with heart failure with NYHA functional class III that rapidly progressed to functional class IV, despite the treatment. HES is a potentially fatal disease, with a survival rate of less than 50% after 10-year follow-up. There are several predictors of early mortality that includes intraventricular conduction delay, duration of symptoms prior to presentation, NYHA functional classes III and IV, and the presence of an embolic event. Our patient had two of these early mortality predictors (NYHA functional class IV and pulmonary embolism) and rapid deterioration; finally he deceased [10, 11].

Echocardiographic and radiological studies could be a useful tool in determining cardiac anatomy and function; however, Löffler endocarditis requires a pathological diagnosis; therefore endocardial biopsy remains the gold standard. Nevertheless, in some cases the cardiac biopsy could be a risky procedure; therefore the clinician should assess the inherent risk of this intervention in each particular clinical setting. In addition, it is indispensable to rule out Löffler endocarditis when diagnosis of pulmonary disorders associated with hypereosinophilia is considered. Additionally, it is important to discard the main differential diagnosis of HES when assessing the possibility of Löffler endocarditis, which includes hypereosinophilia secondary to hypersensitivity reactions and parasite infections [4].

In this case, despite the endomyocardial biopsy result, the patient had peripheral hypereosinophilia and typical echocardiographic findings of restrictive cardiomyopathy; therefore the diagnosis of Löffler endocarditis was established and then was confirmed during autopsy. Pathological finding in Löffler endocarditis includes fibrous thickening of the endocardium, leading to apical obliteration, thrombus formation, and restrictive cardiomyopathy, which clinically manifest as heart failure, thromboembolic event, and atrial fibrillation [5–7].

HES treatment primary goals are to reduce eosinophil level in peripheral blood and tissue, preventing end-organ damage and avoiding adverse thrombotic events. Heart failure in Löffler endocarditis is mainly due to diastolic rather than systolic dysfunction; therefore treatment includes intravenous diuretics to decrease cardiac preload [4]. In addition, for the treatment of symptomatic patients, such as this case, the first-line drug of choice is corticosteroids followed by cytotoxic agents such as hydroxyurea or immunomodulatory agents such as interferon-alpha. Glucocorticoid treatment resulted in clinical and biopsy-proven improvement of eosinophilic and myocardial damage as well as normalization of peripheral hypereosinophilia [12, 13]. Other recent therapeutics includes tyrosinase inhibitors and new types of monoclonal antibodies (Imatinib) [4, 14]. The patient received glucocorticoid treatment without favorable response; his heart failure continued to worsen and led to his death within one week.

## 4. Conclusion

Löffler endocarditis is a rare entity probably underdiagnosed and underreported worldwide and, in Hispanic populations, this pathology represents a diagnosis challenge for the attending physician. Therefore, when HES is suspect, an echocardiographic study should be indicated with the intention of determining if there is a restrictive pattern, and if this pattern is present, a biopsy is indicated. When there is a high clinical suspicion of HES and image studies that support the possibility of Löffler endocarditis and early mortality predictors are present, we consider that treatment should be initiated immediately even in the absence of a definitive pathological diagnosis.
